# Tibial eminence fractures in the paediatric population: a systematic review

**DOI:** 10.1007/s11832-014-0571-6

**Published:** 2014-03-02

**Authors:** Christy Coyle, Simond Jagernauth, Manoj Ramachandran

**Affiliations:** Department of Trauma and Orthopaedic Surgery, Royal London Hospital, Whitechapel Road, London, E1 1BB UK

**Keywords:** Paediatric tibial eminence, Tibial spine, Anterior cruciate ligament avulsion, Fracture

## Abstract

**Introduction:**

We present a systematic review of the literature for the management of tibial eminence fractures in the paediatric population. Our aims were to assess modalities of injury, treatment options available and their associated complications.

**Materials and methods:**

We found 740 relevant citations in the English literature up to 1 October 2012, of which 36 full text articles met our inclusion criteria.

**Results:**

Our results show that skiing, sports and motor vehicle accidents are increasingly common modes of injury, in addition to the commonly described fall off of a bicycle. Most studies advocate non-operative management for type I Meyer’s and McKeever’s fractures and reduction and internal fixation for type II and III fractures. Better long-term results have been reported with arthroscopic surgery compared to open surgery. There is no consensus as to which type of fixation is best suited for tibial eminence fractures; methods available include excision of the bony fragment, K-wire, screw and, absorbable suture fixation, and more recently, suture anchor and meniscal arrow. The main complications reported include arthrofibrosis, non-union, mal-union, pain and severe laxity. Early post-operative range of motion exercises have been shown to reduce the incidence of arthrofibrosis.

**Conclusion:**

As all papers report results from small case series, Level I studies are required to produce more definitive evidence for the management of paediatric tibial eminence fractures.

## Introduction

Fractures of the tibial eminence represent avulsion fractures of the anterior cruciate ligament (ACL) insertion [1]. They are uncommon, with an age peak in children and adolescents [2–8]. It is equivalent in aetiology to mid-substance ruptures of the ACL in adults [1, 6, 8, 9]. With stress, the incompletely ossified tibial eminence in the child fails before the ligament through the cancellous bone beneath the subchondral plate [3, 10, 11]. Often the fracture extends into the weight-bearing portion of the articular surface of the medial tibial plateau [10, 11].

**Fig. 1:**
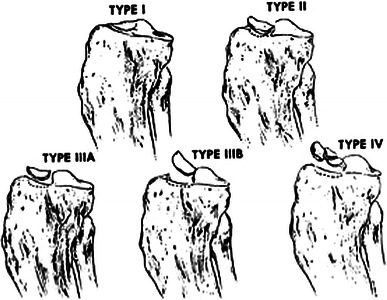
Modified Meyers and McKeever classification according to Zaricznyj [13]

Meyers and McKeever classified these injuries in 1959 as non-displaced (type I), partially displaced or hinged (type II) and completely displaced (type III) fractures [12]. Type III fractures were further subdivided into ‘not rotated’ and ‘rotated’. This classification was modified by Zaricznyj [13] to include comminuted avulsion fractures (type IV) (see Fig. 1).

Non-operative treatment of type I tibial eminence fractures is recommended by most authors [7–10, 14]. The management of type II and III fractures, however, remains controversial [1, 4, 5, 7, 10]. There is no consensus in the literature regarding closed versus open treatment, type of internal fixation and post-operative management [1, 5, 7, 15]. We performed a systematic review of the literature in order to formulate evidence-based recommendations for the management of tibial eminence fractures in the paediatric population.

## Materials and methods

**Fig. 2:**
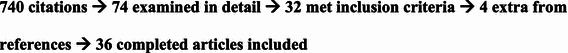
Flow diagram showing the identification of relevant articles

The databases examined were MEDLINE (Medical Literature Analysis and Retrieval System Online), CINAHL (Cumulative Index to Nursing and Allied Health Literature, Ipswich, Massachusetts), EMBASE (Excerpta Medica Database, Amsterdam, The Netherlands), the Cochrane Library without date restriction, and the search engine Google Scholar. The keywords and medical subject heading (MeSH) terms used were ‘tibial eminence fracture’ ‘tibial spine fracture’, ‘anterior cruciate ligament (ACL) avulsion’ and ‘pa(e)diatric anterior cruciate ligament (ACL)’. The articles were selected in three stages. First, the title and abstract of all citations identified by the above searches were downloaded and the list was narrowed using the inclusion/exclusion criteria in Table 1. Secondly, full texts of this shortlist were retrieved and evaluated for eligibility. The most common reason for exclusion at the second stage was because the results for adult tibial eminence fractures were indistinguishable from those for paediatric tibial eminence fractures. If the results were distinguishable, only the paediatric population was included. Thirdly, reference lists of reviewed papers were searched for any additional relevant articles and were also reviewed. Two independent reviewers (CC and SJ) examined the citations with regard to their eligibility, and any doubts were resolved by discussion. Figure 2 indicates how the final papers were selected.

**Table 1:** Inclusion and exclusion criteria

*Inclusion*
Randomised studies
Observational retrospective studies
Controlled clinical trials
Meta-analyses
English language articles
Age group 2–18
*Exclusion*
Case series <4 patients
Studies not separating adult and paediatric population
Review articles
Case reports: technical studies
Series excluding patient involvement
ACL injury without fracture
Non-union or mal-union

Information from the articles was stored in a spreadsheet and the methodology of each was discussed. A descriptive summary of the results is presented here.

## Results

### Epidemiology

This is summarized in Table 2. Several studies included adults and children; to reconcile this, only separated data for the paediatric population was included [4, 6, 15–19]. Although these fractures tend to occur in late childhood and early adolescence, the age range varied from 3 to 18 years old. There was no differentiation in the literature between a younger and an adolescent age group. Traditionally, falling off of a bicycle has been thought to be the main mode of injury [3, 5, 11, 19–26]. More recently, increasing modes of injury for tibial eminence fractures have been reported, including sport, motor vehicle accidents (MVA) and skiing [1–6, 20–25, 27, 28].

**Table 2:** Mean age and modalities of injury leading to tibial spine avulsions in the paediatric population

Paper	Total no.	Mean age	Male to female ratio	Mechanism of injury				
				Bicycle	Skiing	MVA	Sports	Other
1	6	12	2:4	1	4	0	1	0
2	31	12	9:22	–	–	16^a^	–	–
3	20	9	8:12	9	0	5	3	0
4	32	12	19:13	–	–	–	–	–
5	43	12	20:23	4	27	1	5	2
6	14	13	10:4	–	–	–	–	–
7	5	15	3:2	–	–	–	–	–
8	15	12	14:1	1	0	0	1	3
9	10	14	7:3	4	3	0	3	0
10	26	16	16:10	0	0	10	5	11
11 and 34	45	11	36:9	14	0	5	0	9
14	61	–	–	–	–	–	–	–
15	4	12	2:2	0	0	0	4	0
16	8	11	6:2	0	8	0	0	0
17	6	12	5:1	–	–	–	–	–
18	4	11	4:0	0	0	0	4	0
19	47	–	25:22	24	0	10	6	7
20	10	15	9:1	1	0	0	9	0
21	17	12	9:8	5	0	2	5	0
22	12	11	5:7	2	10	0	0	0
23	8	13	2:6	3	5	0	0	0
24	50	13	41:9	20	0	7	15	8
25	41	12	–	–	–	–	–	–
26	11	12	4:7	–	–	–	–	–
27	32	12	18:14	0	8	5	8	11
28	9	13	1:8	0	0	1	8	0
29	80	12	45:35	0	0	4	76	0
30	14	12	12:2	3.5^b^	0	3.5^b^	6	1
31	40	11	26:14	–	–	–	–	–
32	10	–	–	–	–	–	–	–
33	12	13	11:1	3.5^c^	0	4	3.5^c^	1
35	6	14	4:2	–	–	–	–	–
37	15	–	–	–	–	–	–	–
38	8	–	6:2	–	–	–	–	–
40	14	13	8:6	–	–	–	–	–
Totals	*n* = 766	12.4	1.5:1	95 21.1 %	65 14.5 %	73.5 16.4 %	162.5 36.2 %	53 11.8 %

^a^Denotes other mechanisms of injury not mentioned in article

^b^Seven cases attributed to either fall off bicycle or MVA

^c^Seven cases attributed to either sports or fall off bicycle

The types of studies and their outcome methods are presented in Table 3. Except for two prospective case series, all other studies were retrospective case reviews. There was a varied method of reporting outcome, including clinical follow-up only, outcome scores and objective tests. The most common outcome scores were the International Knee Documentation Committee (IKDC), the Tegner Activity Score (TAS) and the Lysholm Knee Score (LKS) [1, 5, 6, 9, 10, 14–16, 20, 21, 23, 27, 30, 32, 35]. The most common objective test was the KT1000 for measuring knee laxity [1, 6, 7, 9, 15, 21, 22, 24, 26–28, 32, 35].

**Table 3:** Types of studies indicating methods of outcome and follow-up data

Paper	Type of study	Methods of outcome			Mean follow-up (years)	Follow-up range (years)
		History, clinical exam (CE), Xray (XR)	Outcome scores	Objective tests		
1	Retrospective case series		IKDC^a^, TAS^b^, LKS^c^, Marshall knee score	KT1000	3.2	2.0–6.3
2	Retrospective case series	History, CE	–	–	15.0	5.0–29.0
3	Retrospective case series	History, CE, XR	–	–	8.8	2.0–16.0
4	Retrospective case series; multicentre	CE, XR	–	–	Not specified	0.5–2.0
5	Retrospective case series	History, CE, XR	IKDC^a^, Cincinatti Knee Score	Hop test, MRI	3.5	1.0–7.5
6	Retrospective case series	CE	LKS^c^	KT1000	3.7	1.8–9.0
7	Retrospective case series	History, CE, XR	–	KT1000	8.5	7.5–9.0
8	Retrospective case series	History, CE, XR	–	–	1.0	0.5–3.0
9	Retrospective case series	CE, XR	IKDC^a^, TAS^b^, LKS^c^	KT1000	7.2	1.7–15.7
10	Retrospective case series	CE	LKS^c^	–	Not specified	2.0–8.0
11 and 34	Retrospective case series	History, CE, XR	–	Genucom knee testing (navigation)	Not specified	3.0–10.0
14	Retrospective case series; multicentre	History, CE, XR	TAS^b^, LKS^c^	Knee signature system, dynamometer	16.0	10.0–39.0
15	Prospective case series	History, CE	IKDC^a^	KT1000	Not specified	≤1.0
16	Retrospective case series	–	IKDC^a^, TAS^b^, LKS^c^	–	2.7	1.2–4.3
17	Retrospective case series	Operative paper only reporting on irreducible type III fractures			Not specified	Not specified
18	Retrospective case series	History, CE, XR	–	–	Not specified	Not specified
19	Retrospective case series	History, CE	–	–	Not specified	Not specified
20	Retrospective case series	CE, XR	IKDC^a^	–	3.5	0.8–6.5
21	Retrospective case series	CE	LKS^c^	KT1000	3.0	0.5–7.0
22	Retrospective case series	CE	–	KT1000	3.0	Not specified
23	Retrospective case series (separate cadaveric study also)	CE	IKDC^a^		1.4	0.5–2.5
24	Retrospective case series; multicentre	History, CE	–	KT1000	4.0	2.0–8.0
25	Retrospective case series	History, CE, XR	–	–	14.2	0.8–22.0
26	Prospective case series	History, CE, XR	–	KT1000	Not specified	3.0–10.0
27	Retrospective case series	–	IKDC^a^	KT1000, Dynamometer	9.7	2.0–16.0
28	Retrospective case series	CE, XR	–	KT1000	3.5	2.1–6.4
29	Retrospective case series	CE	Rate of soft tissue interposition		Not specified	Not specified
30	Retrospective case series	History, CE, XR	IKDC^a^	–	29.0	12.0–42.0
31	Retrospective case series	CE	If arthrofibrosis: MRI ± arthroscopy		1.6	0.3–7.0
32	Retrospective case series; second look surgery for complications	XR	IKDC^a^, TAS^b^, LKS^c^	KT1000, Dynamometer	Not specified	≥6.0
33	Retrospective case series	History, CE, XR	–	Stryker knee laxity tester, Dynamometer	2.6	0.3–2.2
35	Retrospective case series	XR	TAS^b^, LKS^c^, American Knee Society Score, visual analogue score	KT1000, Rolimeter	5.0	3.0–6.0
37	Retrospective case series	History, CE, XR	–	–	7.0	2.5–10.0
38	Prospective case series	History, CE	–	–	1.0	Not specified
40	Retrospective case series; radiological study	–			2.5	0.5–6.0

^a^International Knee Documentation Committee

^b^Tegner Activity Score

^c^Lysholm Knee Score

The distribution of the type of fracture represented in the literature is presented in Table 4. This does not represent the true incidence of the different types in the population, as inclusion criteria for several studies only included type II and III tibial eminence fractures. Most type IV fractures were included with type III fractures; ten papers included the Zarincznyj modification separately [2, 15, 18, 20, 21, 27, 31, 33, 35, 40].

**Table 4:** Types of fracture and methods of management in chronological order of publication

Year of paper	Paper	Fracture type				Management of Type II, III and IV fractures		Non-operative (number)	Operative (number)	
		I	II	III	IV	Type II	Type III ± IV		Open	Arthroscopic
1970	19	12	25	10	^a^	24 non-operative 1 converted to type III and operative	8 operative	38	9	0
1984	25	5	16	20	^a^	All non-operative (includes 7 closed reductions)	8 non-operative (includes 2 closed reductions) 12 operative (5 open reductions only)	29	12	0
1984	37	<3		12	^a^	Type I and II managed together non- operatively	All operative	3	12	0
1986	2	9	11	7	4	Not specified		26	5	1
1987	40	Not specified				Not specified; radiological study		3	11	0
1988	3	3	5	11	^a^	4 non-operative 1 operative	8 non-operative 3 operative	15	4	0
1990	34 and 11	8	14	23	^a^	13 non-operative 1 operative	7 non-operative 17 operative	27	18	0
1992	33	5	5	2	0	2 non-operative 2 operative 1 converted to type III and operative	Operative only	7	5	0
1993	24	3	17	30	^a^	15 non-operative 2 operative	14 non-operative 16 operative	32	18	0
1995	38	0	8		^a^	Operative cases only		0	0	8
1995	14	8	28	25	^a^	All non-operative	12 non-operative 13 operative	48	13	0
1995	32	0	0	10	^a^	Only type III included (operative and non-operative)		7	0	3
1998	28	0	0	9	^a^	Only operative type III included		0	0	9
1999	8	0	0	7	^a^	Only operative type III included		0	7	0
2001	18	0	0	4	0	Only operative type III included		0	0	4
2002	23	0	6	2	^a^	Only operative type II and III included		0	0	8
2002	10	0	16	10	^a^	Only operative type II and III included		0	0	26
2002	17	0	0	6	^a^	Only irreducible type III included		0	0	6
2003	29	0	23	57	^a^	Only operative type II and III included		0	9	71
2003	22	0	0	12	^a^	Only operative type III included		0	12	0
2003	1	0	0	6	^a^	Only operative type III included		0	0	6
2004	16	0	4	4	^a^	Only operative type II and III included		0	0	8
2004	7	0	0	5	^a^	Only operative type III included		0	5	0
2008	21	0	17	0	0	Only operative type II included		0	17	0
2008	6	0	0	14	^a^	Only operative type III included		0	Not specified	
2009	35	0	2	3	1	Only operative types II, III, IV included		0	0	6
2009	5	14	13	16	^a^	All non-operative		43	0	0
2009	9	0	3	7	^a^	Only operative type II and III included		0	0	10
2010	27	8	17	5	2	11 non-operative 6 operative	III and IV all operative	14	18	0
2010	20	0	5	4	1	Only operative type II and III		0	0	11
2010	30	4	3	7	^a^	2 non-operative 1 operative	1 non-operative 6 operative	7	1	6
2010	4	0	25	7	^a^	Only operative type II and III		0	4	28
2011	15	0	2	1	1	Only operative type II, III, IV included		0	0	4
2011	26	0	11		^a^	Only operative type II and III included		0	0	11
2012	31	5	12	20	3	7 non-operative 5 operative	2 non-operative 18 operative (Type III) Type IV all operative	9	2	24

^a^Did not differentiate type IV from type III

### Drainage of haemarthrosis

Haemarthrosis drainage was advocated for both tense haemarthroses for symptomatic relief [2, 19, 20] and to aid in closed reduction for patients managed non-operatively [22, 24, 28, 29, 33]. No case series reported infection after drainage of haemarthroses.

### Entrapment of soft tissue

It is well recognized that the anatomic reduction of tibial eminence fractures can be hampered by the interposition of the anterior horn of the medial or lateral meniscus, or the transverse ligament [1, 16, 20, 22, 27–31]. One case series reviewed 80 fractures that underwent arthroscopic or open reduction for type II or III fractures that did not reduce in extension [29]. They demonstrated entrapment of the anterior horn of the medial meniscus in 36 patients, the anterior horn of the lateral meniscus in one patient, and the transverse ligament in six patients. In the remaining 37 patients, adequate reduction was not obtained despite haemarthrosis aspiration. The authors postulated that smaller tibial eminence fragments theoretically would not have contacted the femoral condyles or trochlea during extension, thereby affecting the reduction. They also discussed that previous authors suggested that trochlear impingement, and not femoral condyle shape, resulted in reduction of the intercondylar eminence [29]. This was confirmed in an arthroscopic study of tibial eminence fractures, which demonstrated that tibial spine fractures did not make contact with the femoral condyles at any stage [32].

A number of smaller case series also reported interposition of menisci or the transverse ligament in the fractures site on arthroscopy [1, 5, 7, 16, 20, 22, 23, 30, 31]. In one case series, a non-union of a type III fracture treated in plaster was attributed to soft tissue interposition [5].

### Fixation versus no fixation

Most studies recommend cast immobilization for the management of type I tibial eminence fractures [7–10, 14, 24, 27]. The debate between non-operative versus operative management of type II and III fractures remains controversial [5–11, 14, 16, 19, 24, 27, 29–34]. Table 4 demonstrates the method of management of different fracture types in chronological order of paper publication. The trend for managing type II, III and IV fractures with reduction and fixation has increased in the last ten years [6, 9, 16, 20–24, 26–29, 35]. Reasons for this include restoring the stabilizing function of the ACL, retrieving interposed soft tissue from within the fracture site, eliminating any mechanical obstruction to motion, reducing the fracture through the medial tibial plateau and potentially decreasing the period of immobilization to minimize stiffness [4, 7, 10, 19, 21, 28–30, 32, 36].

Several older studies have proposed that type II, III and IV fractures do not require fixation [3, 11, 23, 24]. Table 4 outlines differences in management of these fractures patterns. Several papers have reported worse results for type III fractures treated non-operatively [3, 14, 30, 32]. A retrospective case study of 61 paediatric patients found a direct correlation between fracture displacement after healing and knee laxity (*r* = 0.74, *p* < 0.001) [14]. They recommended reduction and internal fixation of all type III fractures.

The most recent study advocating treatment of all tibial eminence fractures non-operatively was published in 2009 [5]. All patients were taken to theatre for closed reduction using an image intensifier. At final follow-up, there was one non-union requiring re-operation, and seven patients reported pain, although none of the patients reported giving way. This is a higher complication rate when compared with other case series that treat type II, III and IV fractures with reduction and fixation, which report no pain and return to sport from a mean of two to five years post operatively [9, 11, 15, 21, 22, 26, 30].

One study demonstrated no difference in their population of type II and III paediatric tibial eminence fractures who were treated with either closed or arthroscopic reduction and a long leg cast (29 patients) versus open reduction and internal fixation (ORIF) (18 patients) [24]. However, ten patients demonstrated a positive pivot shift test at follow-up, but were not identified as to which treatment group they belonged.

One study advocated operative management for type II fractures that did not reduce in extension [16]. In their series of 59 type II fractures, 23 were managed non-operatively as the fracture reduced in extension. Of the 26 type II fractures that did not reduce, only six had interposition of soft tissue. An early series by Meyers and McKeever, however, reported conversion of a type II fracture to a type III fracture with attempted closed reduction [19]. This has also been reported in a more recent series [25].

Another series recommended internal fixation and aggressive rehabilitation for type III tibial eminence fractures, after performing a knee arthroscopy on patients with type III fractures for other morbidities [32]. The patients who had been treated with arthroscopic reduction and internal fixation had less sagittal laxity, increased muscle strength and improved function when compared with the other patients who were treated with closed or arthroscopic reduction without fixation.

### Excision

In a unique approach to the management of type II and III tibial eminence fractures, one paper reported results of arthroscopic excision of the displaced bony fragment if there was decreased range of motion of the affected knee [15]. In their series of seven patients (including four children), arthroscopic excision of the anterior portion of the bony fragment was performed; the posterior footprint of the ACL insertion was normal in all patients and left alone. All paediatric patients returned to their initial level of activity, had full ROM and had less than 5 mm of laxity when compared with the opposite knee. The only other paper to report results after excision demonstrated no instability after open excision in four patients [19]. One paper disputed that only the anterior portion of the ACL was involved, and used a classification system in which type A was the anterior portion only, and type B was a fracture of the entire ACL footprint [3]. Advocates of excision discuss the benefits of early weight bearing and mobilisation [15].

### Arthroscopic versus open reduction

Table 4 demonstrates the increasing popularity of arthroscopic fixation in modern times. Before the regular use of arthroscopy, open reduction for displaced tibial eminence fractures was advocated [2, 8, 11, 19, 34, 37]. Arthroscopic treatment has been reported to result in decreased morbidity, earlier mobilization, and shorter hospital stay [1, 4, 7, 18, 20, 26, 28–30]. Arthroscopic anterior cruciate ligament guides have been popular in aiding in reduction and guiding fixation with arthroscopic techniques [1, 7, 14, 26, 30]. Some techniques include arthroscopic assisted surgery, and report little increased morbidity with a mini-arthrotomy [23, 28].

The most recent case series of open reduction and internal fixation included 17 patients with type II fractures [21]. They demonstrated an average Lysholm score of 99.7 at an average of three years follow-up, with no instability measured with the KT1000.

### Type of fixation

Most authors agree on either arthroscopic or open reduction and fixation for displaced tibial eminence fractures in current practice [4, 7, 10, 14, 18, 19, 21, 22, 24, 26–32, 36]. There is no consensus in the body of literature regarding the best type of fixation. As there are no randomized controlled trials comparing any of these methods, we present only a description of the current literature available.

#### Screws versus wires versus sutures

The most popular fixation devices in the literature are sutures, K-wires and screws [1, 6, 8–10, 14, 18, 20, 22, 28, 30, 33, 38, 39]. Advocates for suture fixation report that screw and K-wire fixation are not suitable for comminuted fractures, and can even cause comminution of the fracture fragment [1, 30, 39]. Investigators have reported full function and return to sport for all patients treated by arthroscopic suturing, with early mobilization and full ROM [1, 22, 28, 30, 33]. Investigators using K-wire fixation have had similar successful results, with patients having no pain or instability at 12 months [20, 38]. This technique can also be physeal sparing, which is safer for the paediatric population [20]. If left proud, K-wires can be removed in the outpatients department, but there may be an infection risk [38].

Traditionally, cannulated partially threaded cancellous screws have been used to fix larger fragments. However, these screws are often removed due to the risk of anterior impingement, fretting between the washer and screw, and potential damage to the articular surface [10, 16, 35]. In addition, this technique is not effective for cases in which a small or a comminuted tibial spine fragment occurs [1]. In one case series of 26 type II and III fractures treated with cannulated screw and washer, four patients had anterior impingement due to ‘ledging’ of the fracture, and two of these patients needed ACL reconstructions within three years of their original surgery [10]. Although epiphyseal screws are used, transphyseal screws are sometimes required for more purchase in the bone [4].

Use of headless screws with differential threads and a smaller diameter (i.e., Accutrack screws and Herbert screws) have also been reported [6, 18]. In a series of four paediatric patients, all patients with fixation using this screw had normal return to sport and function at six months [18].

#### Absorbable versus non absorbable

Absorbable materials avoid complications secondary to hardware or a second operation to remove metalwork [4, 6, 30]. However, in one retrospective study assessing 14 paediatric patients with type III tibial eminence fractures treated with ORIF, children fixed with an absorbable suture had a median increase in laxity of 2 mm compared to 1 mm in children fixed with non-absorbable materials (screw or stainless steel wire loop) [6]. The difference between these two groups, however, was not statistically significant (*p* = 0.36 and *p* = 0.21).

More recently, other devices to fix tibial eminence fractures have been used, such as meniscal arrows or suture anchors [21, 26]. Meniscal arrows have a diameter of 1.1 mm and, therefore, can be used for small fractures fragments and young patients [26].

### Methods of immobilization and rehabilitation

Descriptions for rehabilitation often only included the immediate postoperative period. Few papers describe rehabilitation protocols in detail, and some did not specify a rehabilitation protocol at all [6, 17, 25, 32, 37, 40]. Most authors immobilized fractures if managed non-operatively in an above knee cast, for three to 12 weeks, in a position of a range of hyperextension up to 45° of flexion [2, 3, 5, 11, 14, 19, 24, 27, 30, 33, 34, 38]. One paper recommended casting in hyperextension to prevent arthrofibrosis [5]. In their series of 43 patients treated non-operatively despite the McKeever type, all patients were immobilized in hyperextension for three weeks and flexion for three weeks. No patients lost extension when compared to the other side, although two patients lost 20° of flexion.

Most post operative protocols were similar to the non-operative protocols, with immobilization in extension to 30° of flexion for four to six weeks [1, 3, 4, 7–9, 14, 16, 19, 21, 22, 24, 26, 27, 29, 30, 38]. Only six authors allowed early range of motion (after two weeks or less) after internal fixation of the fragment [10, 15, 28, 30, 31, 35]. Continuous passive motion machines were used in three of these papers from day 1 post operatively [15, 18, 30].

Apart from immobilization, rehabilitation was heterogeneous and not well described in the literature. Weight bearing protocols varied from non-weight bearing for six weeks to weight bearing as tolerated immediately postoperatively [1, 2, 7, 9, 10, 15, 16, 20, 30, 35]. Closed chain quadriceps and hamstrings exercises were started from day 1 in five papers [6, 15, 20, 26, 35]. Physiotherapy and full strength exercises were allowed at four to six weeks by five authors [1, 7–9, 30]. Time to return to sport was also varied. Two authors allowed full sporting activities when range of motion and muscle strength was equal to the contralateral side [7, 15]. Four other authors described time periods ranging from four weeks to five months [9, 16, 29, 35].

### Stability

The most common methods of measuring stability were clinical tests, anterior drawer test, Lachman test and pivot shift, which are difficult to quantify. Of these clinical tests, the pivot shift was the most predictive of clinical instability [10, 30]. One group described a radiological Lachman test to compare post-treatment laxity between the injured and non-injured knee [40]. In their series of 12 patients, they found a mean of 3.34 mm greater active subluxation with the injured knees post-treatment, and consistently found a difference between the injured and normal knee.

Several papers have used the KT1000 to assess laxity more accurately [1, 6, 7, 9, 15, 21, 22, 24, 26–28, 32, 35]. Increased laxity in the affected knee of up to 5 mm has been reported, but these patients had no symptoms of instability [1, 6, 9, 15, 25, 26, 30, 32]. One study reported increased laxity in type II and III fractures of 3.5 mm when compared with the uninjured knee, regardless of closed reduction or open and internal fixation [11]. It is thought that the ACL fibres are elongated at the moment of trauma [1, 9, 10, 21, 25, 37]. The lack of clinical instability is postulated to be due to the integrity of the nerve fibres along the ACL ligament and neuromuscular feedback [9].

In a case series of ten patients, KT1000 measurements of 6 mm (three patients) were associated with a positive pivot glide test [9]. None of these patients had symptoms of instability at follow-up, and all patients returned to their pre-injury sports activity. One paper reported a type III fracture that was treated non-operatively with severe symptoms of instability [30]. On assessment, the patient had a side-to-side difference of 6 mm with the KT1000 and a positive Lachman and pivot shift test. This patient went on to have an ACL reconstruction six years post operatively.

Arthroscopic reduction and internal fixation of type III tibial spine fractures in skeletally immature patients results in persistent laxity but excellent functional outcome [1, 27].

### Assessment of reduction

An X-ray was the most common method of assessing reduction in the acute and longer follow-up period (see Table 3). No authors reported using computed tomography (CT) scans; one group specifically stated their reason for not using CT scans was due to the radiation risk [35]. Magnetic resonance imaging (MRI) was used in one series, assessing ACL integrity and chondral damage rather than reduction [5]. Another series used MRI as a tool for investigating decreased range of motion post operatively [31].

One series reported their results from ten type III fractures that required a second look arthroscopy for other reasons [32]. Patients who had been fixed internally had a lower incidence of mal-union and chondromalacia patellae. Another series of 26 patients used only arthroscopic cannulated screws for fixation of type III fractures [1]. At the time of screw removal, a second arthroscopy revealed no mal-union of the fracture site.

### Complications

The main complications reported in the current body of literature include arthrofibrosis, non-union, mal-union, pain and severe laxity [4–6, 8, 10, 11, 25, 27, 31, 33, 34]. Most small case series report no complications [1, 7, 9, 20, 22, 23, 28, 30, 38].

A retrospective study of 32 tibial avulsion fractures at a mean of 14 years demonstrated 70 % of patients reported pain [27]. This is the highest pain rate reported, and also the longest follow-up time.

One paper reported on a series of 32 patients who developed arthrofibrosis of the knee post-operatively [4]. Of 205 patients with type II or III fractures treated operatively in four centres, 10 % developed arthrofibrosis. 24 patients had arthroscopic lysis of adhesions followed by manipulation, and eight patients had manipulation under anaesthesia (MUA) alone. Of these patients with only closed reduction, the distal femur was fractured during the MUA in three patients, two of which then went onto growth arrest and angular deformity. Therefore, MUA only in conjunction with arthroscopic lysis of adhesions was recommended.

One retrospective review investigated 40 tibial eminence fractures treated with closed reduction [31]. Those who started ROM rehabilitation later than four weeks of treatment were 12 times more likely to develop arthrofibrosis than those who started before four weeks (*p* = 0.029). A lack of full extension of the knee has been associated with a fair to poor result [6].

Early range of motion would, therefore, seem to be a factor in the prevention of arthrofibrosis.

In one series of ten patients who went back for second look arthroscopy up to four years post operatively, mal-union was observed in type III fractures managed without fixation [32]. This same paper reported a higher incidence of chondromalacia patellae in type III fractures managed non-operatively.

Non-union is reported rarely after tibial eminence fractures [5, 6, 25, 39]. In a series of 43 fractures treated non-operatively, there was one case of non-union due to soft tissue interposition [5]. Another study followed up 32 patients at a mean of 14.2 years, and four type III fractures and one type II fracture had a non-union [25]. It is thought that non-unions may be due to insufficient reduction or soft tissue entrapment [5, 21]. Non-unions can be asymptomatic [33].

Although avoiding damage to growth plates is a main concern in ORIF of paediatric intercondylar fractures, there were no reports of growth arrest in the current review reported, except after fracture during MUA for post operative arthrofibrosis [5].

### Long-term outcomes

Several papers describe outcomes of tibial eminence fractures greater than fiv e years after the injury [2, 3, 7, 9, 14, 25, 27, 30, 37] (see Table 3). Most series with long-term follow-up were treated before the advent of arthroscopic surgery, and pain after sport and some loss of extension was considered a ‘good’ result [3, 25, 27, 30]. Thirty-one patients with a mean of 15 years follow-up demonstrated that type III fractures treated with either closed reduction or ORIF had the worst prognosis when compared with type I and II fractures [2]. In another study of 14 paediatric patients, with a mean follow-up time of 29 years, the best results were observed in patients treated with arthroscopic reduction and fixation [30]. The worst result was observed in one patient with a type III lesion treated non-operatively, who had severe symptoms of instability and had an ACL reconstruction six years after the injury.

Since the advent of arthroscopic reduction and improvements in fixation, long term results have improved [21]. In ten patients treated with arthroscopic reduction and fixation followed up at a mean of seven years, all were able to return to pre-injury levels, and had a full range of motion [9]. In another recent series, patients with type III fractures treated with arthroscopic reduction and successful fixation treatment were able to resume their pre-injury activities at a mean of 8.5 years post-injury [7].

### Limitations

The purpose of the study was to perform a systematic review of the literature of the epidemiology and management of tibial eminence fractures in the paediatric population. Unfortunately, there are no level one studies at the present time on this topic, so the conclusions are based solely on the case series presented in the literature. In addition, there are widely varied reports of principles of treatment, treatment methods, outcome measures, rehabilitation and length of follow-up. Due to the retrospective nature of most case series, these factors within the same paper were often varied. Nine of the case series reported findings from clinical follow-up alone [2–4, 8, 18, 19, 22, 25, 37, 38]. Other papers focussed specifically on surgical technique or complications, and did not mention a minimum length of follow-up or follow-up technique [17–19, 29, 31].

This systematic review, therefore, highlights the need for level one trials with modern techniques of reduction and fixation.

On review of the current literature, we would overall support arthroscopic or open reduction and internal fixation for irreducible type II and type III fractures. With newer arthroscopic and fixation techniques, the benefits include restoring the ACL, retrieving interposed soft tissue, decreasing the risk of mal-union and potentially decreasing the period of immobilization to minimize stiffness. It should be noted that the data utilised for this review may be skewed by studies from the non-arthroscopic era of literature.

Several series included all types of tibial eminence fractures, regardless of their type or management [2, 3]. However, most differentiated which type II fractures were managed operatively and non-operatively. One series managed all type II fractures with closed reduction, and operated only on those that did not reduce [29]. Unfortunately, not all case series had a consistent management algorithm for type II III and IV fractures, and, therefore, this may introduce bias into their series [2, 3, 11, 14, 24, 27, 30, 31, 34]. However, there was a clear outline of which types were managed operatively and non-operatively in most series, which is outlined in Table 4.

Most of the literature on outcomes of tibial eminence fractures focus on patient reported outcome measures and knee laxity, rather than muscle strength. Due to the small numbers in the case series, it is difficult to draw any conclusions from results from symptomatic patients.

Not all papers outlined important data such as method of injury or how the patients were recruited [4, 6, 7, 9–11, 14, 16, 17, 25, 26, 29, 31, 32, 34, 35, 37, 38, 40]. This is a limitation of the current review. However, not including these papers would mean a significant loss of important data, such as the largest case series of 80 patients and important information regarding complications [4, 17, 29, 32].

There is significant literature on biomechanical studies that was excluded from this review. This may provide further information on the management of tibial spine fractures in particular; early mobilization regimens and a systematic review of these studies alone may be warranted.

## Conclusions

Although there are no randomized trials focusing on paediatric tibial spine fractures, a number of observations can be made from this systematic review. In the paediatric population, the mean age of injury is in adolescence. Skiing, sport and MVAs are now increasing modes of injury, in addition to the traditional fall off of a bicycle. If closed reduction is impossible in type II or III fractures, this may be due to entrapment of the anterior horn of the lateral or medial meniscus, or transverse ligament.

Arthroscopic or open reduction and internal fixation for type II and III fractures is advocated in the current literature, although some case series of closed reduction of these fractures demonstrate no difference in outcome. With newer arthroscopic and fixation techniques, the benefits of surgery include restoring the ACL, retrieving interposed soft tissue, decreasing the risk of mal-union and potentially decreasing the period of immobilization to minimize stiffness.

There is no consensus as to the type of fixation that is best for tibial eminence fractures; methods available range from excision to K-wire to screw to absorbable sutures, or more recently suture anchors or meniscal arrows. Complications and return to theatre related to metalwork can be avoided by using absorbable materials; however, there may be a tendency to increased laxity of the knee. Arthrofibrosis may be avoided by early mobilization. Treatment of arthrofibrosis should include arthroscopic division of adhesions to avoid complications of MUA. Emerging long-term results favour better outcomes with arthroscopic surgery compared with open surgery.

It should be noted that all studies on this topic are case series, often with small numbers of patients. Further prospective research, with larger numbers and assessing more modern techniques of treatment, is required.
